# Post-endodontic pain evaluation after different intracanal laser assisted disinfection techniques. A Systematic Review

**DOI:** 10.4317/jced.59941

**Published:** 2023-02-01

**Authors:** Haitham Elafifi-Ebeid, Pablo Betancourt, Isabel Parada-Avendaño, Josep Arnabat-Domínguez

**Affiliations:** 1 Faculty of Medicine and Health Sciences, University of Barcelona, 08907 Barcelona, Spain; 2 Research Centre for Dental Sciences (CICO), Department of Integral Adult Dentistry, Universidad de La Frontera, Temuco 4810296, Chile; 3 Analysis and Design in Clinical Investigation, University of Barcelona, 08017 Barcelona, SpainAnalysis and Design in Clinical Investigation, University of Barcelona, 08017 Barcelona, Spain; 4 Idibell Institute, 08908 Barcelona, Spain

## Abstract

**Background:**

Post-endodontic pain (PEP) management is an important factor to be considered in endodontic treatment. Several risk factors have been described that can attribute to its appearance. Laser-assisted disinfection has been described by many authors for its antimicrobial effect. Few studies described the relation between laser disinfection and its effect on PEP. The objective of this review is to describe the relation between different intracanal laser disinfection techniques and their effects on PEP.

**Material and Methods:**

An electronic search strategy was performed in Pubmed, Embase, and Web of Science (WOS) databases without restrictions as to the date of publication. Eligibility criteria were randomized controlled clinical trials (RCT) that used one of the different intracanal laser disinfection techniques in their experimental groups evaluating PEP outcome were included. Risk of bias analysis was performed by the Cochrane risk of bias tool.

**Results:**

The initial research identified 245 articles from which 221 were excluded and 21 studies were sought for retrieval and 12 articles met our inclusion criteria for the final qualitative analysis. The laser systems used were Nd:YAG, Er:YAG and, diode lasers including photodynamic therapy.

**Conclusions:**

The diode lasers showed the most promising results in terms of PEP reduction while Er:YAG showed more short-term efficacy (6 hours postoperative interval). The variables could not be analyzed homogenously due to the differences in the study designs. More RCT are needed comparing different laser disinfection techniques with the same baseline endodontic pathology to establish a specific protocol for the best outcome.

** Key words:**Root canal treatment, Post-endodontic pain, Intracanal laser disinfection, laser dentistry.

## Introduction

One of the main goals of root canal treatment is to manage endodontic pain. Post endodontic pain (PEP) after treatment can last from 24 to 72 h (1,2).

Nagendrababu *et al*. (3) described risk factors associated with PEP such as age range from 41 to 65 years old, being female, mandibular molars, presence of preoperative pain, debris and microorganisms extrusion beyond tooth apex during instrumentation, absence of preoperative periapical radiolucency, inadequate local anesthetic choice, high concentrations of sodium hypochlorite (NaOCl) during irrigation, warm vertical compaction obturation technique, traumatic occlusion of the treated tooth, and lack of operator experience.

Several studies (4-7) focused on investigating different treatment protocols to study their effect on PEP such as the rotary file system used, concentration of NaOCl, activation approaches of the irrigants and obturation system in terms of the type of sealer used and its periapical tissue biological response, but results are still contradictory.

Various laser wavelengths have been investigated in the endodontic field not only for their antimicrobial efficacy (8) but also for the anti-inflammatory modulation and smear layer removal. All these advantages add more benefits to root canal therapy (9,10).

Every specific wavelength used has different target tissue absorption, which can be called chromophore or pigment. It is described that the neodymium-doped yttrium aluminum garnet (Nd:YAG) (wavelength range 1064-1440nm) laser is one of the first laser systems investigated as an adjunctive therapy to root canal treatment due to its ability to melt and resolidify the dentinal walls, which seems to reduce dentine permeability, improving the sealing of the root canals (11,12), although another study found no significant difference in apical sealing ability (13). The Nd:YAG laser also proved to be more effective in removing pigmented bacteria (14).

Diode lasers are highly absorbed in melanin and hemoglobin and they have greater penetration capacity inside the root canal walls, which can be beneficial for deeper antimicrobial effect acting on pigmented bacteria (15). They can also reach distant areas such as the periapical zone producing a photochemical effect and reducing inflammation, accelerating healing and achieving analgesia (16).

Laser activated irrigation (LAI) has been studied in endodontic irrigation protocols with the medium infra-red erbium: yttrium aluminum garnet (Er:YAG) (wavelength 2940nm) and erbium, chromium:yttrium scandium gallium garnet (Er,Cr:YSGG) (wavelength 2780nm) lasers because they are highly absorbed in water. Their mechanism of action takes place when the water molecules absorb light energy, which leads to a microexplosion generating strong photomechanical shock waves that can remove the smear layer from root canal walls. This phenomenon is called photon-induced photoacoustic streaming (PIPS) (17). This technique is considered safe in terms of the apical extrusion of irrigants. Arslan *et al*. (18) found no difference in the extrusion generated by PIPS compared with conventional and ultrasonic irrigation. Moreover, lower concentrations of NaOCl (0.5%) activated for 60s proved to be equally effective in root canal disinfection compared to higher concentrations (2.25%), which can reduce the complications in case of accidental irrigant extrusion (19).

Photodynamic therapy is another alternative to enhance the antimicrobial effect during root canal treatment. Its mechanism of action is based on the interaction between a photosensitizer (PS) and its compatible wavelength in the presence of oxygen molecules releasing highly reactive oxygen singlets (1O2) which cause microbial cell damage (20). The power settings needed ranges from 40-100mW which makes it safer to use, avoiding possible complications like thermal damage to the surrounding tissues that can take place with diode or Nd:YAG lasers when improperly used (8).

The objective of this systematic review is to describe the effect of different laser root canal disinfection techniques on PEP through the qualitative analysis of randomized controlled clinical trials to provide clinicians with more information regarding if there is an added value for the patients when using lasers as an adjunctive tool in root canal disinfection.

## Material and Methods

We reviewed the literature through an electronic search strategy in Medline (PubMed), Embase and Web of Science (WoS) databases without restrictions as to the date of publication till the year 2021. We performed an advanced search in PubMed as (endodontic treatment OR root canal treatment OR root canal therapy OR endodontics) AND (Laser OR phototherapy OR laser therapy) AND (Postoperative pain OR pain OR postoperative complications).

We also searched Embase by introducing the keywords (‘endodontics’/exp OR endodontics) AND (‘laser’/exp OR laser) AND (‘postoperative pain’/exp OR ‘postoperative pain’) and finally a search strategy in Web of Science as TS= (endodontics or root canal treatment) AND TS= (laser or phototherapy) AND TS= (pain or postoperative pain or postoperative complications).

All articles were screened through the title and abstract, and we only chose randomized controlled clinical trials related to intracanal laser treatment and its effect on postoperative pain for full review. We also performed a manual search to ensure we included all relevant articles. Two independent authors performed the search, study screening and selection, and there was no disagreement concerning study selection.

-Inclusion criteria.

• Articles in English

• Randomized controlled clinical trials with a control or placebo group.

• The intervention group carried out intracanal irradiation using one of the following lasers (Er:YAG, Er,Cr:YSGG, diode, Nd:YAG or photodynamic therapy).

• Conventional endodontic treatment performed in all groups.

• Endodontic treatment performed in single or multiple visits.

• Studies evaluating the pain variable by VAS or NRS

• Studies that included human permanent teeth with the following characteristics:

▪ Mature apex

▪ Primary endodontic infection with vital or necrotic pulps

▪ Secondary endodontic infection (retreatment cases).

-Exclusion criteria:

• Case reports or case series, narrative review, letter to the editor or short communications and non-randomized clinical trials or pilot studies.

• RCT that used additional photobiomodulation application after treatment.

• Primary dentition or permanent teeth with immature apex.

• *In vivo* animal studies or *in vitro* studies.

**Figure 1:**
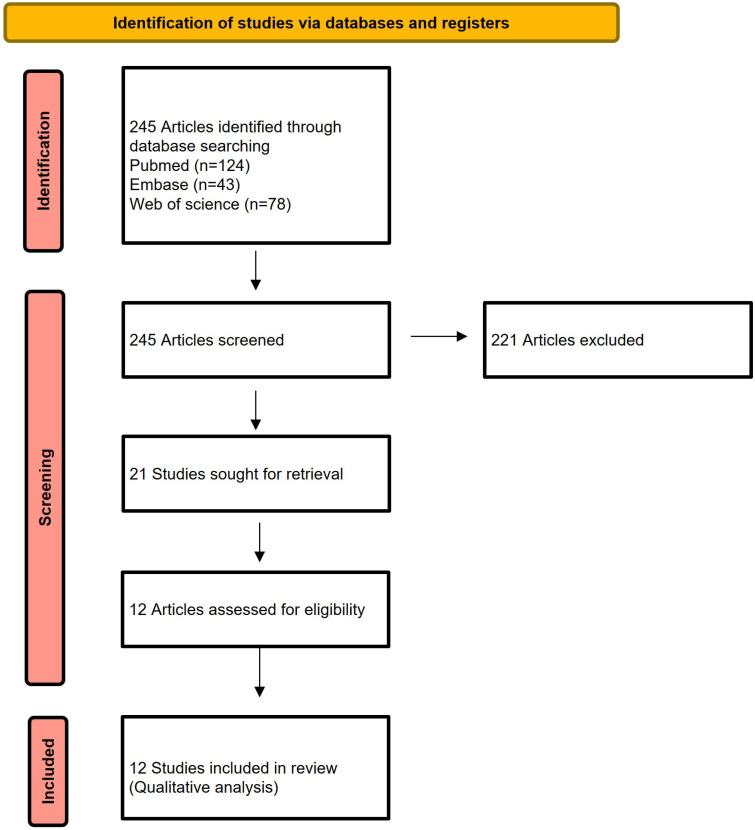
Prisma flow chart.

Study selection and inclusion according to the PRISMA flow chart (Fig. 1).

-Eligibility criteria:

This systematic review was conducted according to the Preferred Reporting Items for Systematic Review and Meta-Analysis (PRISMA) guidelines. We focused on the following question: Does the literature, to date, provide a relation between a specific laser assisted intracanal disinfection technique and its effect on PEP reduction?

The population, intervention, comparison, outcomes (PICO) process was used to answer the previously focused question.

Population: Patients with primary or secondary endodontic infection.

Intervention: One of the following intracanal laser assisted disinfection technique (Er:YAG, Nd:YAG, or diode laser including the photodynamic therapy).

Comparison: Conventional endodontic chemo-mechanical disinfection with lack of intracanal laser disinfection or placebo laser (if applicable).

Outcomes: Less PEP in the intervention groups from a minimum of 6 h to a maximum of 2 weeks.

**Figure 2:**
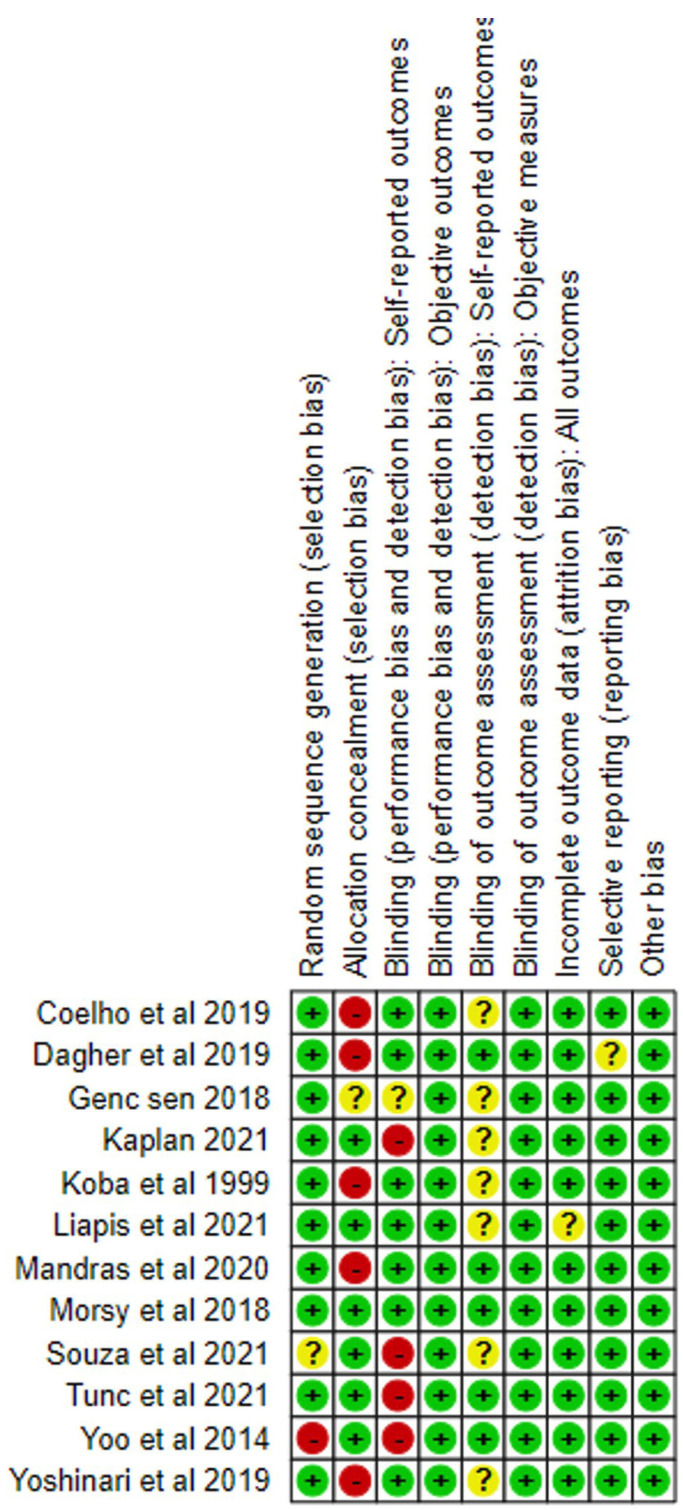
Risk of bias graph: review authors’ judgments about each risk of bias item presented as percentages across all included studies.

Risk of bias analysis:

We performed the quality assessment of individual studies using the Cochrane risk of bias tool (21) (Fig. 2).

## Results

After study screening and duplicates removal we found 124 articles in PubMed, 43 articles in Embase and 78 articles in web of science. Only 12 articles met the inclusion criteria and were eligible for full review and final qualitative analysis (Fig. 1).

Outcome evaluation: We evaluated post operative pain as a primary outcome in intervention groups with different types of intracanal laser disinfection and the control or placebo groups.

The reviewed studies used the following laser systems in their experimental groups: Nd:YAG laser (22-24), diode lasers (22,25-27), Er:YAG laser activation (28-30) and aPDT (31-33). There was no study using Er,Cr:YSGG laser that met the inclusion criteria.

Regarding the baseline endodontic pathology, 10 studies included primary endodontic infection (23-25,27,28-33) 3 of them reported the presence of symptoms at the beginning of the treatment (22,28,29), and 2 articles included secondary endodontic pathology (23,26).

Seven studies included cases with necrotic pulps (24,25,27,29,31-33) and 4 authors described the presence of signs of radiographic periapical pathology (27,28,32,33).

Relevant data were extracted from the included studies such as author, year of publication, study design, study groups, age, endodontic pathology, treatment visits, pain evaluation methods, minimum duration without medication intake before treatment, number of analgesics needed after treatment, postoperative pain evaluation time intervals and outcomes ( Table 1). Laser type and parameters from every study were also extracted according to the laser system used ( Table 2- Table 4).

**Table 1:**
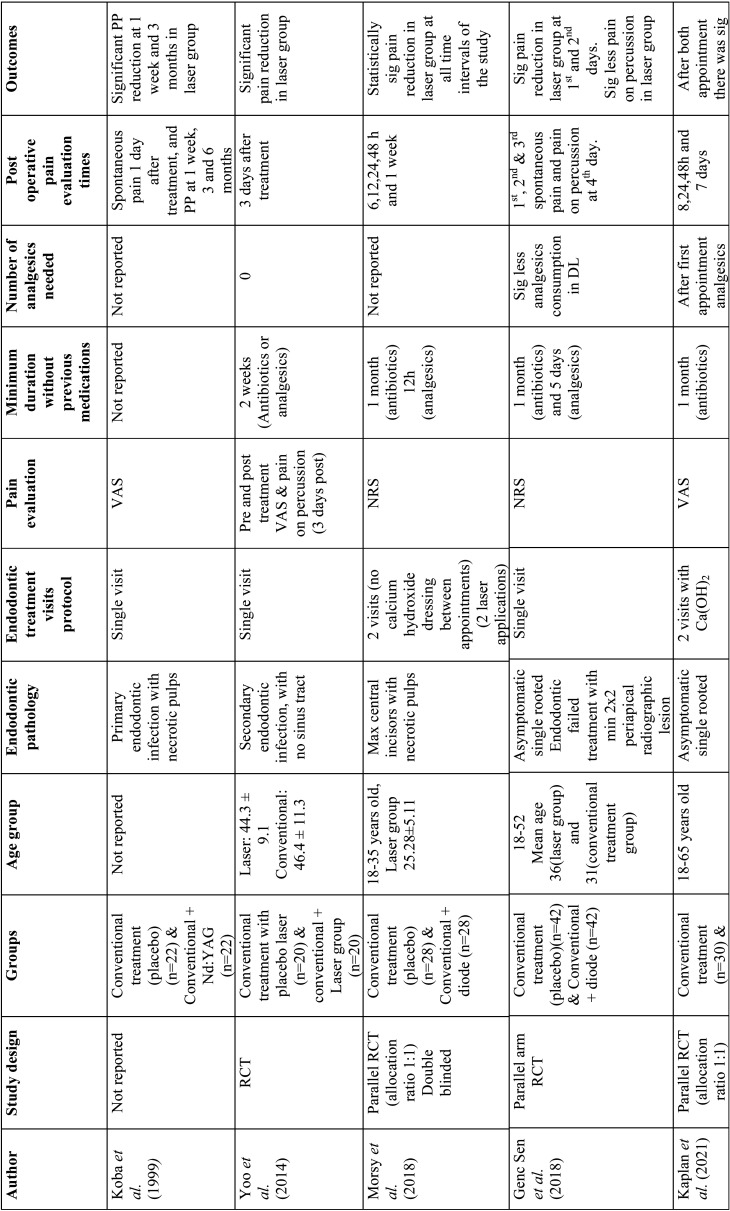
Data extraction from included studies.

**Table 1 cont.**
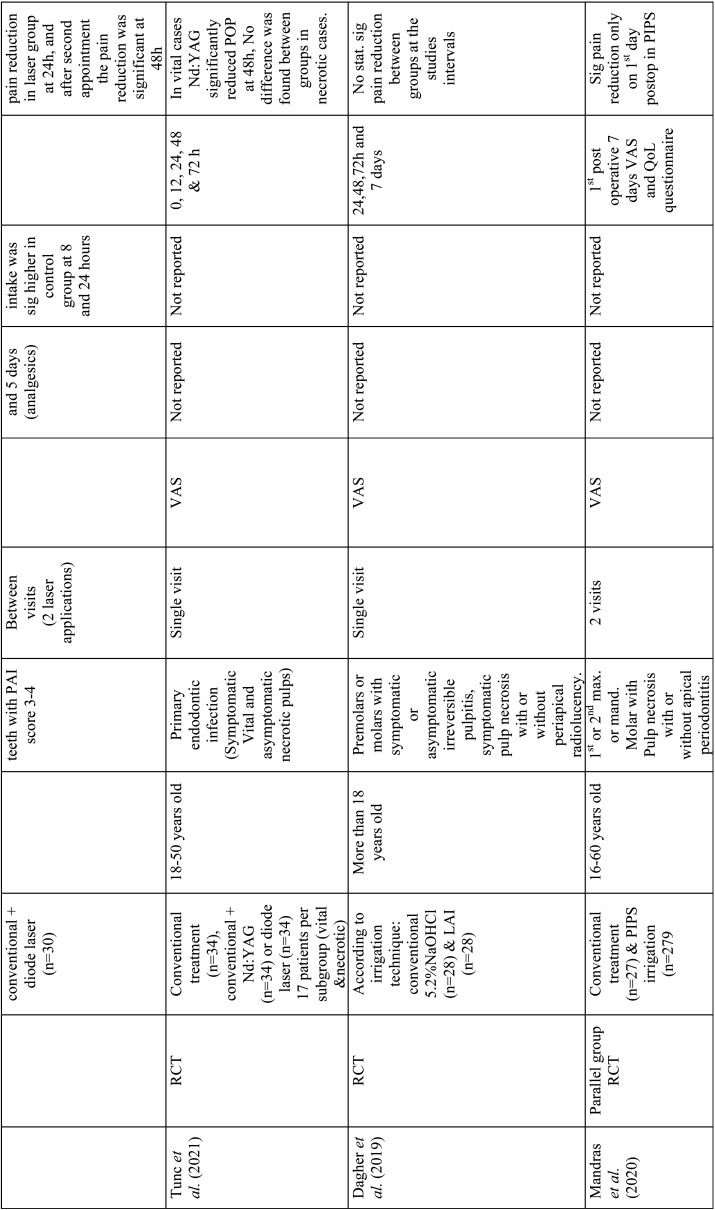
Data extraction from included studies.

**Table 1 cont.-1:**
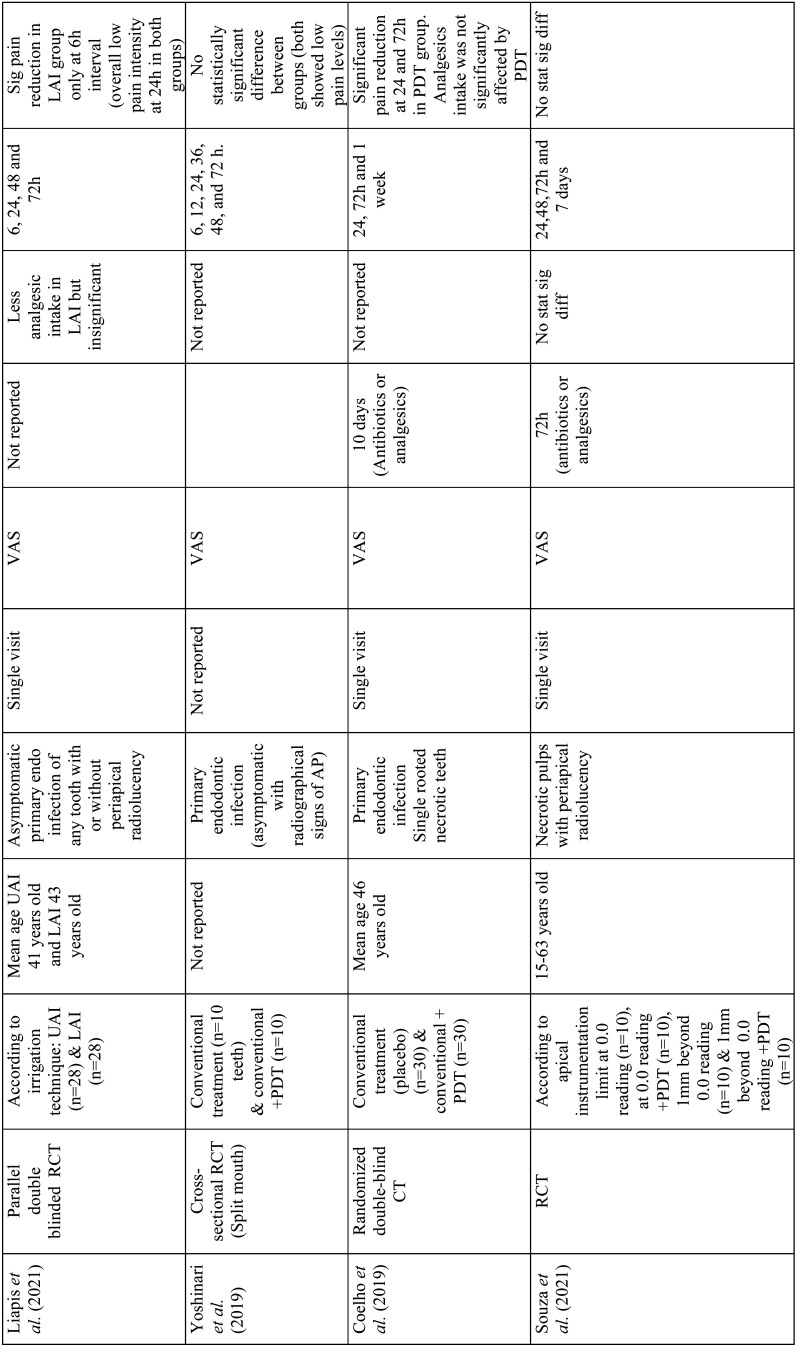
Data extraction from included studies.

**Table 2:**
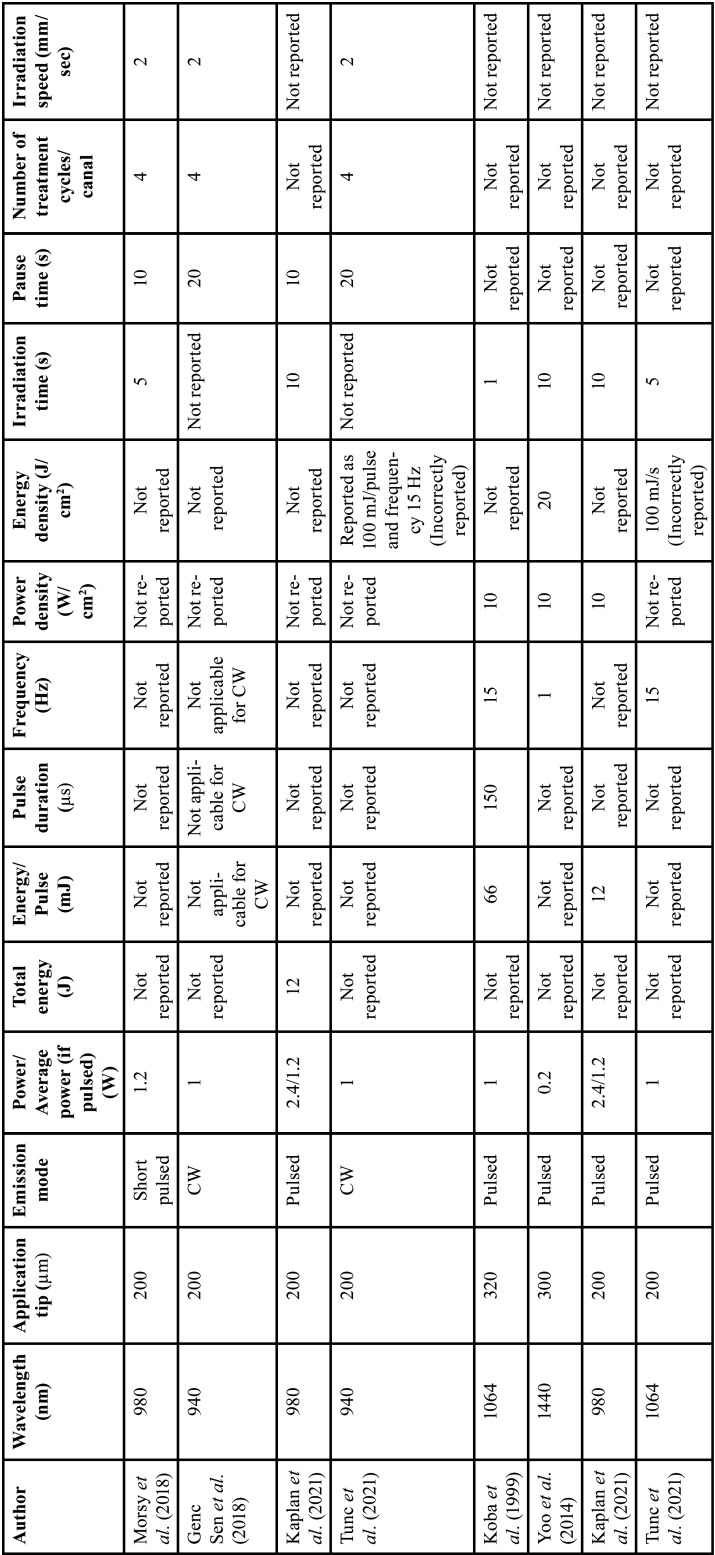
Nd:YAG and Diode laser reported parameters.

**Table 3:**
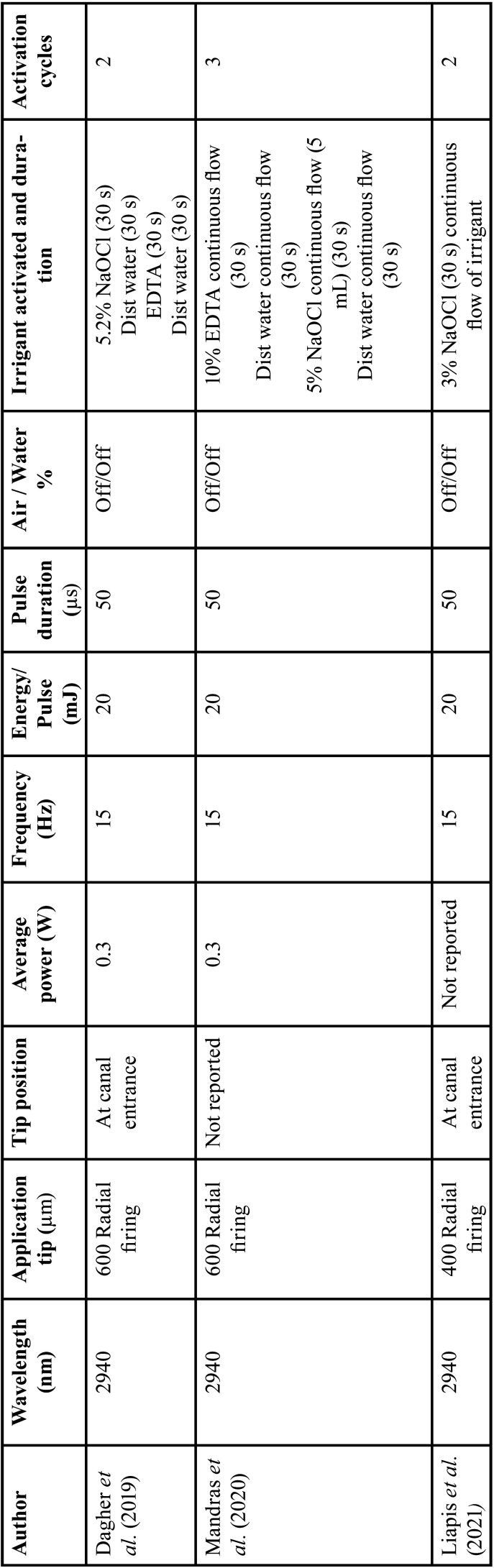
Er:YAG reported laser parameters.

**Table 4:**
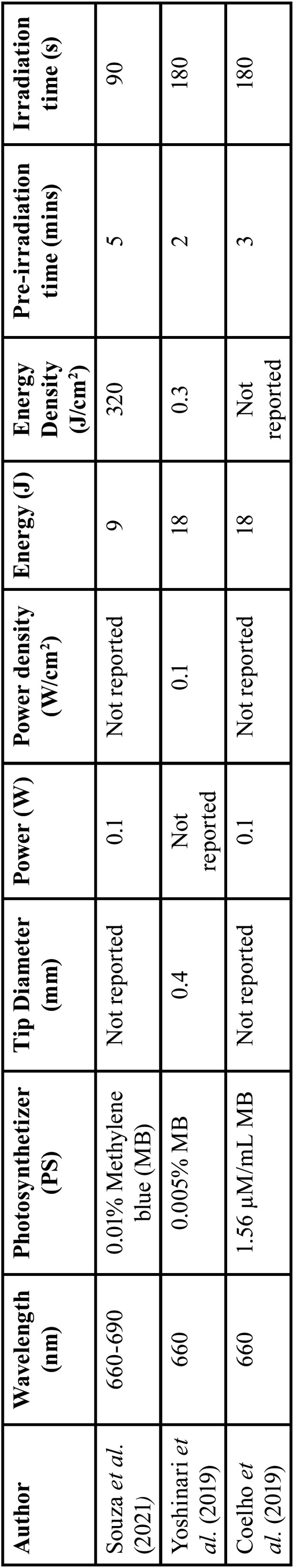
PDT reported parameters.

Risk of bias analysis resulted in 7 studies with moderate (22-24,30,30-32) and 5 studies with low risk of bias (25-27,29,33) (Fig. 2).

Regarding the pain evaluation method 10 studies used the VAS (22-24,27,28-33) and the remaining 2 studies chose the NRS (25,26).

## Discussion

Postoperative pain is one of the most frequently studied topics in endodontics, because it directly affects the patient´s quality of life and is the main cause for patients to seek treatment (34).

The presence of bacteria has been described as the main cause of postoperative pain in the literature (35), although authors such as Mandras *et al*. (29) reported through a randomized clinical trial that there was no direct correlation between the presence of remanent bacteria and pain symptoms. This shows that it is not entirely clear whether it is enough for only one of these factors to be present or whether the combination of more factors is necessary for the appearance of pain (30).

One of the main drawbacks for measuring the degree of pain is its subjectivity. The VAS is among the most used pain measurement tools (22,27,28). It should be noted that each patient has a unique pain threshold, which makes it difficult to compare results through the application of a VAS (23). Yoo *et al*. (23) correlated the degree of pain measured by VAS with the quantification of levels of inflammatory cytokines and neuropeptides of the inflammatory exudate after applying a Nd:YAG laser. The results showed that the level of perceived pain decreased significantly in the laser group; however, the level of cytokines and neuropeptides did not reach the value “0”, in any of the measurements, not even in the patients with absence of pain. Despite these discrepancies, the VAS scale remains widely accepted due to its ease of understanding and high reproducibility (36).

The included studies that used Er:YAG reported the same laser parameters in relation to average power, energy per pulse, frequency, air and water percentage. However, there was a heterogenicity concerning the activation cycles parameter. Two studies performed 2 activation cycles, one of them combined EDTA with NaOCl (28), while the other only used NaOCl (30). Conversely, Mandras *et al*. (29) performed 3 cycles alternating EDTA with NaOCl.

The authors evaluated postoperative pain at different time intervals. Mandras *et al*. (29) found significant pain reduction in laser groups at 24 h, while Liapis *et al*. (30) reported better results only at 6 h. Dagher *et al*. (28) reported no significant difference between groups, which is not consistent with these studies. These differences in outcomes could be attributed to the difference in the baseline endodontic pathology of the patients and different teeth groups.

Concerning the diode laser, two different wavelengths were reported in the included studies (940 and 980nm). The authors who applied the 980nm (25,27) selected pulsed power settings with an average power of 1.2W.

Both authors performed the endodontic treatment in 2 sessions. Morsy *et al*. (25) found significant pain reduction at all postoperative time intervals (6,12,24,48 h and 1 week), whereas Kaplan *et al*. (27) reported better results only at 24 h after the first session and at 48 h after the final visit. The baseline endodontic pathology was the same in terms of pulpal state (necrotic pulps) but the second author (27) included teeth with a periapical score index between 3-4 which is less likely to suffer PEP in all groups.

The 2 authors who used 940 nm (22,26) applied continuous emission mode with a power of 1W. Genc sen *et al*. (26) found statistically significant differences in spontaneous pain during the first and second postoperative days, including less pain on percussion at one week, while Tunc *et al*. (22) found no statistically significant differences in the diode laser groups. There was also heterogenicity between the experimental groups, the first author (26) included secondary endodontic pathology while the latter (22) included only primary endodontic infections, which may explain the discrepancy in the obtained results.

Concerning the Nd:YAG lasers, the 1064nm wavelength was used by 2 authors (22,24) with the same average power of 1W, whereas Yoo *et al*. (23) used the 1440nm wavelength with an average power of 0.2W.

All 3 authors found significant pain reduction in the laser groups, but at different time intervals studying patients with different baseline endodontic pathologies. Tunc *et al*. (22) found differences only in vital cases and no differences among necrotic ones. By contrast, Koba *et al*. (24) found statistically significant differences at 1 week and 3 months in necrotic cases. Yoo *et al*. (23) found significant pain reduction in secondary endodontic pathology at 3 days interval.

The studies using PDT reported very similar parameters, such as wavelengths, PS and power settings, with some differences in pre-irradiation and irradiation times. Among the 3 studies, only 1 study (32) found significant pain reduction at 24 and 72 h treating necrotic teeth with no previous signs of periapical radiographic pathology, while the other 2 authors (31,33) found no statistically significant pain reduction in the laser groups. This can be attributed to their inclusion of patients with preoperative radiographic signs of periapical radiolucency, which is less likely to have PEP in either groups.

Concerning analgesic intake, Genc sen *et al*. (26) reported an average number of analgesic pills over 3 days in the control group (1.11 ± 2.14) compared to those in the laser group (0.11 ± 0.52). Kaplan *et al*. (27) compared the analgesic intake by the patients in both groups after the first and second visits at intervals of 8, 24 and 48 h. They found that after the first visit 40% of patients in the control group needed analgesics at 8 h and 23.3% at 24 h compared to 0% in the laser group. After the second visit only 6.7% of patients needed analgesics at 8 h compared to 0% in laser group.

Both studies indicated that the differences were statistically significant concerning less analgesic consumption in the laser group at the mentioned time intervals.

## Conclusions

• From all the included studies the diode laser showed the most promising approach in terms of postoperative pain reduction, which may be due to its deeper tissue penetration, reaching the periapical tissues causing inflammatory modulation and an additional analgesic effect.

• The pain reduction in the case of Er:YAG showed short-term efficacy in the first 6 to 24 hours.

• The combination of 2 different techniques is still unknown, but it would be interesting to investigate the possibility of superior results.

• Finally, more randomized controlled clinical trials are needed to compare different laser systems but including the same baseline endodontic pathology and symptoms to avoid bias and demonstrate the best specific technique for PEP reduction.
